# Dosimetric intercomparison for stereotactic radiotherapy of multiple brain metastases

**DOI:** 10.1016/j.phro.2026.100956

**Published:** 2026-03-28

**Authors:** Sara Abdollahi, Rachid Boucenna, Cécile Chatelain, Nathan Corradini, Marie Fargier-Voiron, Vincent Fave, Juan Garcia, Sarah Ghandour, Matthias Guckenberger, Martin Härtig, Tanja Hertel, Maud Jaccard, David Jeller, Stephan Klöck, Krayenbühl Jérôme, Natacha Ruiz López, Peter Pemler, Harald Petermann, Olivier Pisaturo, Francesco Pupillo, Daniel Schmidhalter, Christian Tata, Sheeba Thengumpallil, Sergejs Unterkirhers, Veronique Vallet, Patrick Weber, Nicolaus Andratschke, Stephanie Tanadini-Lang

**Affiliations:** a Department of Radiation Oncology, Universitätsspital Zürich, Zürich, Switzerland; b Department of Radiation Oncology, Hirslanden Clinique Bois-Cerf, Lausanne, Switzerland; c Department of Radiation Oncology, Radio Onkologiezentrum Biel Seeland, Biel-Bienne, Switzerland; d Department of Radiation Oncology, Clinica Luganese Moncucco, Lugano, Switzerland; e Department of Radiation Oncology, Clinique de Genolier, Genolier, Switzerland; f Department of Radiation Oncology, Hôpital de La Tour, Meyrin, Switzerland; g Department of Radiation Oncology, Atrys Schweiz AG, Liestal, Switzerland; h Department of Radiation Oncology, Hôpital Riviera-Chablais, Vaud-Valais, Rennaz, Switzerland; i Department of Radiation Oncology, St. Claraspital AG, Basel, Switzerland; j Department of Radiation Oncology, Kantonsspital St.Gallen, St.Gallen, Switzerland; k Department of Radiation Oncology, Clinique Générale-Beaulieu, Genève, Switzerland; l Department of Radiation Oncology, Luzerner Kantonsspital, Luzern, Switzerland; m Department of Radiation Oncology, Lindenhofspital, Bern, Switzerland; n Department of Radiation Oncology, Hôpitaux Universitaires de Genève, Genève, Switzerland; o Department of Radiation Oncology, Stadtspital Zürich Triemli, Zürich, Switzerland; p Department of Radiation Oncology, Universitätsspital Basel, Basel, Switzerland; q Department of Radiation Oncology, Hôpital Fribourgeois, Fribourg, Switzerland; r Medical Physics Unit, Imaging Institute of Southern Switzerland, Ente Ospedaliero Cantonale, Bellinzona, Switzerland; s Division of Medical Radiation Physics and Department of Radiation Oncology, Inselspital, Bern University Hospital, University of Bern, Bern, Switzerland; t Department of Radiation Oncology, Clinique de La Source, Lausanne, Switzerland; u Department of Radiation Oncology, Hirslanden Clinique des Grangettes, Chêne-Bougeries, Switzerland; v Department of Radiation Oncology, Klinik Hirslonden, Zürich, Switzerland; w Department of Radiation Oncology, Centre Hospitalier Universitaire Vaudois, Lausanne, Switzerland; x Department of Radiation Oncology, Réseau Hospitalier Neuchâtelois, La Chaux-de-Fonds, Switzerland

**Keywords:** Intracranial radiotherapy, Dosimetric intercomparison, Quality assurance

## Abstract

**Background and purpose:**

Stereotactic radiotherapy (SRT) delivers high doses with submillimeter precision to small targets, requiring accuracy in both dose calculation and delivery. This study aimed to perform a dosimetric intercomparison of SRT accuracy across multiple radiotherapy departments.

**Materials and methods:**

The study included 30 SRT plans submitted by 24 hospitals. A head phantom, equipped with Gafchromic EBT3 film and ion chamber inserts, was used for dose measurements. Invited institutions generated SRT treatment plans prescribing 30 Gy in 5 fractions to brain targets, following their clinical protocols. Point dose and planar dose measurements were carried out at each institution, then the measured doses were compared against those calculated by their respective treatment planning systems. Agreement between planned and measured dose was assessed, with gamma passing rates calculated using 5%/1 mm and 3%/1 mm criteria.

**Results:**

An average difference of 0.2% and a range of − 4.1% to 4.3% between the ion chamber measured dose at the center of one of the targets and the calculated dose was found. For the 5%/1 mm global gamma criterion, all films achieved passing rates above 97%. All treatment devices showed sub-millimeter spatial dose delivery accuracy, with deviations under 1 mm between measured and planned dose.

**Conclusions:**

The results indicated clinically acceptable agreement between the calculated and delivered dose across all treatment delivery systems. This intercomparison provided an opportunity for radiotherapy departments to assess the quality of their SRT treatment planning and delivery and benchmark their performance against other institutions.

## Highlights

• Nationwide intercomparison of stereotactic radiotherapy in brain metastases.
• Ion chamber dose differences ranged from – 4.1% to 4.3% across 30 measurements.
• Film measurements showed median gamma pass rates ≥99% (5%/1 mm) and ≥95% (3%/1 mm)
• Film dose profiles agreed within 1 mm with the planned dose profiles of all centers.

## Graphical abstract

**Figure:**
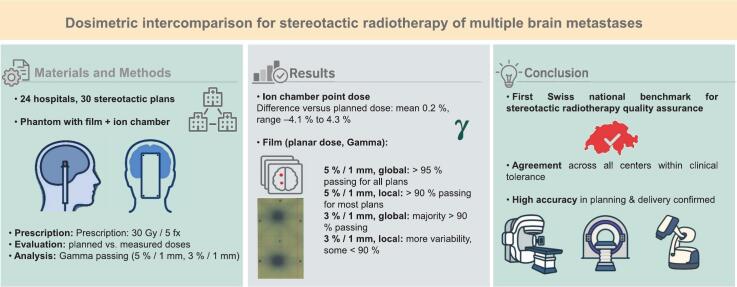

## 1 Introduction

Stereotactic radiotherapy (SRT) is an effective therapeutic option for patients with multiple brain metastases (BM) [1], [2], [3], [4]. Stereotactic radiosurgery (SRS) is increasingly used for the treatment of 4–15 BM [5], [6], [7]. Treating multiple lesions simultaneously is associated with increased complexity compared to a single lesion for several reasons. Employing a multi-plan technique for multiple lesions is time-consuming and prone to setup errors [8]. Moreover, effectively managing the radiation spillage from low doses within the fields optimized by one plan while concurrently optimizing those with another plan poses a challenge. Using a single plan technique increases sensitivity to geometric uncertainties, potentially risking compromise in target dose, especially in the presence of rotational errors, particularly for smaller volume targets or those distant from the isocenter [9], [10], [11]. In addition, dose calculation and MLC positioning accuracy for the multiple off-axial targets in a single plan SRS treatment could pose considerable challenges [12], [13], [14].

Over time, there has been an improvement in technology enabling simultaneous targeting and treatment of multiple intracranial metastases, and recent analyses have shown that this is possible without loss in clinically relevant accuracy and local control [15]. Accurate and reproducible dose delivery to the primary target is therefore considered essential in stereotactic techniques, with recent guidelines emphasizing rigorous quality assurance throughout the treatment chain [16], [17]. Meeting this objective demands consistent attention to quality assurance at each stage, contributing to the final dose calculation, setup based on imaging, and contributions from target and organ delineation, dose calculation, and treatment delivery.

In advanced and complex radiotherapy techniques, such as SRS, characterized by small target volumes, high dose variations, and rapid dose fall-off, any geometric uncertainties can directly lead to dosimetric uncertainties. The effectiveness of this treatment relies heavily on the accuracy of dose calculations and delivery. A companion publication has separately analyzed inter-institutional differences in treatment planning and prescription practices within the same cohort of centers [18].

End-to-end testing is crucial in evaluating the accuracy and efficacy of the SRT workflow, both during the initial commissioning phase and as an integral component of the annual quality assurance program [19]. National intercomparisons offer valuable opportunities to guarantee the safe delivery of radiotherapy and maintain consistency in patient outcomes. It can also enhance standards and accuracy of radiotherapy practices in many healthcare facilities over time [20], [21].

The objective of this study was to perform a nationwide dosimetric intercomparison to evaluate the accuracy and consistency of dose calculation and delivery for stereotactic radiotherapy of multiple brain metastases across radiation oncology departments in Switzerland. This work established a national benchmark for SRT by assessing performance under clinical end-to-end conditions across the major treatment platforms used in the country.

## 2 Materials and methods

**Fig. 1:**
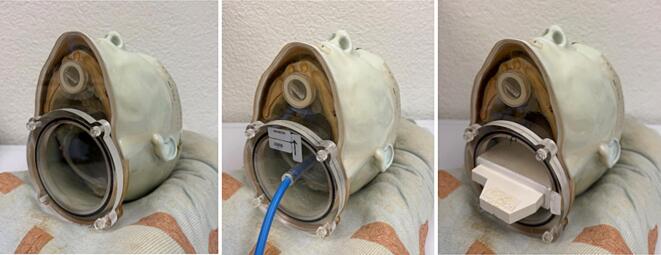
Prime phantom, homogeneous (left) incorporating PinPoint 3D ion chamber (middle), incorporating film (right).

A dosimetric intercomparison was conducted using an anthropomorphic 3D printed head phantom, the RTsafe Prime (RT-safe, Athens, Greece) at the University Hospital of Zurich (USZ). The phantom is an anatomical replica of a real human head, which is filled with water and designed to accommodate Gafchromic EBT3 film (Ashland ISP Advanced Materials, NJ) and a PTW 30016 PinPoint3D ion chamber (PTW, Freiburg, Germany) for planar and point dosimetry measurement. To ensure accurate positioning of the phantom, a custom-designed head support (AccuForm cushion, Civco Medical Solutions) was employed. Three computed tomography (CT) scans were performed on the phantom using a Siemens Somatom Edge Plus CT scanner (Siemens AG, Erlangen, Germany), each with a different phantom configuration: the homogeneous phantom (Primary CT), the phantom with an ion chamber (Secondary CT#1), and phantom with incorporating film (Secondary CT #2), all in a reproducible position (Fig. 1). The scans were acquired using a standard clinical imaging protocol (120 kV, 430 mAs, slice thickness 0.6 mm, medium-smooth Br38 kernel for reconstruction).

**Fig. 2:**
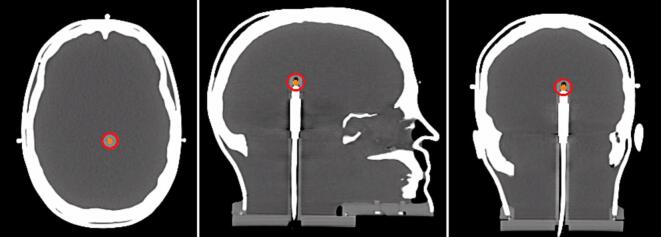
CT scan (secondary scan #1) of the phantom including the PinPoint 3D chamber. The sensitive volume is delineated in orange and the 1.5 cm- PTV in red around the sensitive volume. (For interpretation of the references to colour in this figure legend, the reader is referred to the web version of this article.)

Three CTs were co-registered in Eclipse treatment planning system v16.01.10 (Siemens Healthineers, Germany). The sensitive volume of the PinPoint ion chamber was delineated on the Secondary CT #1 and transferred to the primary CT (Fig. 2). The Central metallic pin on the film cassette was delineated on the Secondary CT #2 and transferred to the primary CT.

Within the brain region of the primary CT, three spherical planning target volumes (PTVs) (1.5 cm, 1 cm, 0.5 cm) and the relevant critical organs (brainstem, chiasm, eyes, optic nerves, lenses) were delineated. The primary CT and structure set were then sent to participating institutions to generate clinical SRT plans prescribing 30 Gy in 5 fractions to all targets, following each center’s routine protocol. A CT Hounsfield-unit–to-density calibration curve was also provided to ensure consistent dose calculation based on phantom materials. All participating centers used a 1 mm dose‐calculation grid for stereotactic treatment planning, consistent with standard SRT practice.

**Fig. 3:**
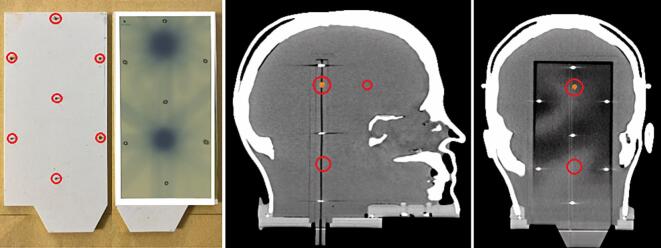
Film Insert and Target Localization within the Phantom. (Left) Irradiated film and EBT3 film insert with seven metal pins (red circles) embodied in a RW3 slab for positioning. (Middle, sagittal view) CT scan (secondary scan #2) showing the three PTVs; with the sensitive volume of the PinPoint chamber centered in PTV1 (highlighted in orange). (Right, coronal view) CT coronal slice showing the inserted film plate within the phantom. (For interpretation of the references to colour in this figure legend, the reader is referred to the web version of this article.)

Thirty SRT plans from 24 departments were analyzed, representing 13 TrueBeam, 5 Edge, and 2 Clinac iX linear accelerators (Linac) (Siemens Healthineers, Germany), 2 Synergy and 1 Versa HD linacs (Elekta AB, Stockholm, Sweden), 5 CyberKnife systems (Accuray Incorporated, Sunnyvale, CA, USA), and 2 Radixact systems (Accuray Incorporated, Sunnyvale, CA, USA) (Supplementary Table S1). In five centers, multiple treatment devices were included. Digital imaging and communications in medicine (DICOM) dose files returned by the centers were imported into a plan with isocenter placed on the film cassette’s central metallic pin in Eclipse. When separate plans were submitted for different targets, dose files were first accumulated in MIM software, v7.1.6 (MIM Software Inc., Cleveland, OH, USA) before import. The coronal planar dose at the film plane was then exported from Eclipse to FilmQAPro software, v7 (Ashland Advanced Materials, Bridgewater, NJ, USA) for comparison with the measured dose (see Fig. 3)

For conciseness, the detailed methodology for ion chamber and film dosimetry is presented in Supplementary Material A. In seven centers, repeated measurements were performed when initial results were unsatisfactory. The reasons included a change in planning or delivery technique (1 case), identifiable human error (3 cases), and unclear causes (3 cases). The description of repeated measurements, including their causes and outcomes, is provided in Supplementary Material B. The statistical analysis used in this study is presented in Supplementary Material C.

## 3 Results

**Fig. 4:**
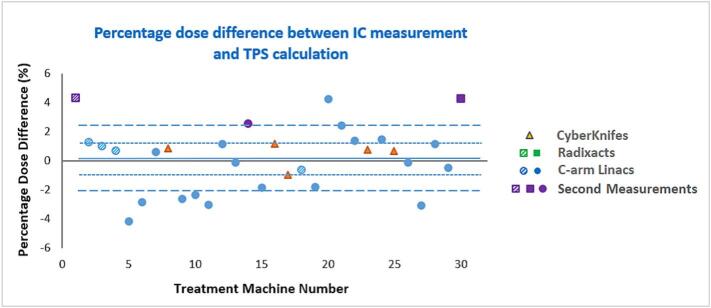
Percentage dose difference between ion chamber measurements and TPS-calculated doses across all treatment machines. Pattern-filled markers indicate systems not commissioned for SRS/SRT, while purple markers denote second measurements performed in three centers. The solid line shows the overall mean difference; dotted and dashed lines represent ±1 and ±2 SDs, respectively. (For interpretation of the references to colour in this figure legend, the reader is referred to the web version of this article.)

The differences between the ion chamber–measured dose and the dose calculated by the treatment planning system (TPS) for thirty measurements ranged from – 4.1% to 4.3%, with an average of 0.2% and a standard deviation of 2.2% (Fig. 4). The C-arm linacs showed a spread in percentage difference of – 4.1% to 4.3%, with an average of – 0.2%. For the two Radixact machines, the repeated (second) measurements showed dose differences of around 4.3%, whereas the initial measurements had been higher (7–8%). CyberKnife systems yielded dose differences within the range of – 1% to 1.2%, with an average of 0.5%.

Fig. S1 in the Supplementary Materials illustrates a representative film–TPS dose comparison. For the 5%/1 mm global criterion, all films exceeded 98% for the superior and 97% for the inferior target; under local normalization, all but one exceeded 96% and 92%, respectively (see Supplementary Fig. S2 and S3 for the corresponding superior and inferior target gamma passing rates for the 5%/1 mm criteria).

**Fig. 5:**
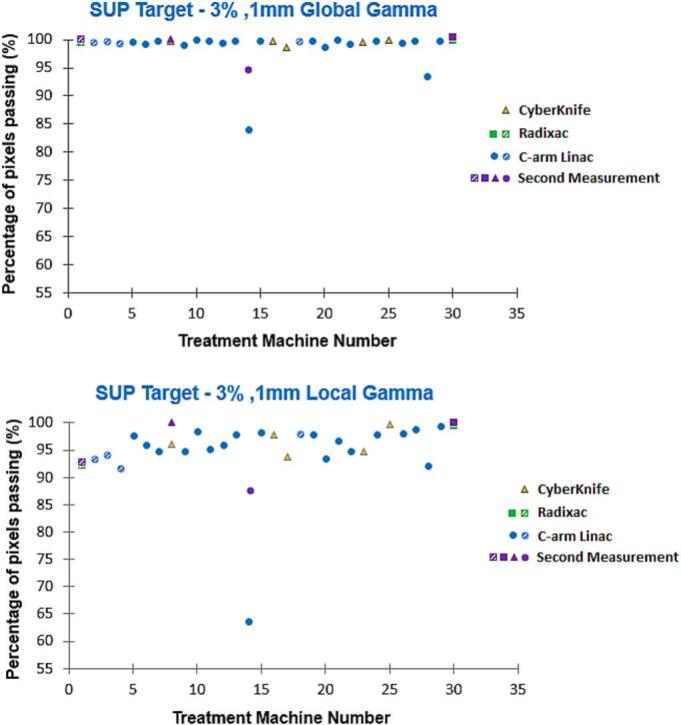
Gamma passing rates for superior target (3%, 1 mm global and local criteria) across treatment platforms. Purple markers denote second measurements, while pattern-filled markers indicate systems not commissioned for SRS/SRT. (For interpretation of the references to colour in this figure legend, the reader is referred to the web version of this article.)

**Fig. 6:**
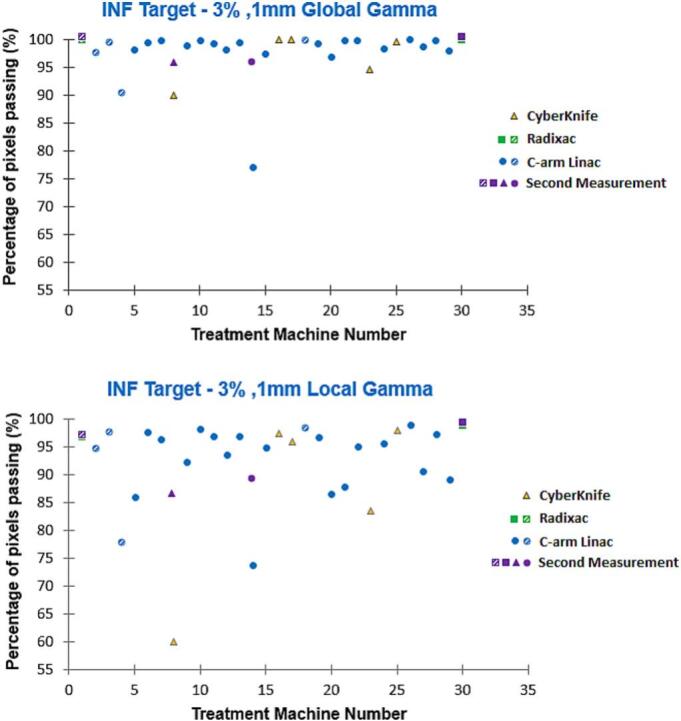
Gamma passing rates for inferior target (3%, 1 mm global and local criteria) across treatment platforms. Purple markers denote second measurements, while pattern-filled markers indicate systems not commissioned for SRS/SRT. (For interpretation of the references to colour in this figure legend, the reader is referred to the web version of this article.)

With the 3%/1 mm global criterion, all but two films surpassed 98% for the superior target, and all but one exceeded 95% for the inferior target. For the 3%/1 mm local criterion, all but one film showed passing rates above 91% for the superior and above 84% for the inferior target. The single outlier for both gamma criteria corresponded to a system not commissioned for SRS/SRT. Fig. 5, Fig. 6 show the gamma passing rates under the 3%/1 mm global and local criteria for superior and inferior targets. Median gamma pass rates were 100% (5%/1 mm global) for both targets, 99.2% and 99.1% (5%/1 mm local), 99.7% and 99.4% (3%/1 mm global), and 96.4% and 95.7% (3%/1 mm local) for the superior and inferior targets, respectively.

In addition to gamma analysis, dose profile comparisons along the in-plane and cross-plane directions consistently demonstrated sub-millimeter agreement (<1  mm) between measured and planned distributions across all participating centers.

## 4 Discussion

This study presents a national dosimetric intercomparison assessing dose calculation and delivery for stereotactic radiotherapy across multiple Swiss centers. Overall, a good agreement was observed between calculated and measured doses across different treatment platforms. Most systems demonstrated consistent performance under clinical end-to-end conditions. A single outlier was observed for a platform that had not been commissioned for SRT, highlighting the importance of appropriate system commissioning for small-field stereotactic applications.

The observed deviation was primarily driven by a reduced local gamma passing rate for the inferior target in this non-SRS system. This center had developed three separate plans for the three targets, and a positional inaccuracy was observed only in the inferior-target plan. As this linac lacked automatic couch correction after cone beam CT (CBCT), the most plausible explanation is a manual error when applying CBCT offsets. This highlights the value of fully automated image-guided workflows for stereotactic radiosurgery [22], [23], [24]. Excluding two repeated measurements attributable to initial human error, the remaining repeated measurements occurred in centers actively engaged in clinical SRT.

A key strength of this study is the decision to allow participating institutions to use their routine clinical protocols rather than a standardized test plan. This design provided insight into the real-world variability and consistency of stereotactic dose calculation and delivery workflows across Switzerland. Protocol differences are recognized contributors to inter-institution variability in multicenter dosimetric audits, as demonstrated in national stereotactic and multi-institution credentialing studies [25], [26]. We also clarified that several repeated measurements were initiated by the centers themselves as part of routine quality assurance (QA) troubleshooting rather than due to methodological issues, reinforcing that the observed variability reflects clinical practice and enhancing the relevance of our findings.

All treatment devices demonstrated sub-millimeter spatial dose-delivery accuracy, with deviations under 1 mm between measured and planned doses. However, gamma passing rate variations were greater for the inferior target than for the superior target in both C-arm linacs and CyberKnife systems. In CyberKnife systems, this was mainly attributable to positional deviations (approximately 0.6 mm lateral and longitudinal). In C-arm linacs, the lower pass rates stemmed from dose deviations, likely related to suboptimal beam modeling in the TPS for the smaller 1 cm^3^ inferior target compared with the larger 2 cm^3^ superior target, which is a well recognized challenge in stereotactic radiotherapy [27], [28].

The C-arm linac group showed the widest spread in percentage differences compared with the CyberKnife and Radixact groups, although overall agreement with the TPS remained good. This broader variability likely reflects the larger number of machines and the diversity of techniques, commissioning procedures, and beam models. In contrast, Radixact and CyberKnife systems exhibited more uniform performance, consistent with their standardized workflows. These findings align with the UK SRS audit by Dimitriadis et al. [25], noting that the standard deviation was smaller here despite a similar maximum deviation of ∼4%.

For both Radixact systems, the ∼4% deviation was reproduced consistently, with ion chamber and film–plan comparisons at the PTV1 center yielding similar results. This pattern suggests a systematic offset rather than random error, likely related to local calibration procedures, small-field dosimetry challenges, and TPS beam modeling under stereotactic conditions. These deviations remain within the expected range for advanced delivery systems but highlight the need for ongoing independent verification in high-precision radiotherapy. At the same time, Radixact systems achieved very high gamma pass rates even at 3%/1 mm, reflecting strong mechanical precision and spatial delivery accuracy. Thus, the consistent ∼4% ion chamber deviation should not be interpreted as reduced dosimetric accuracy but rather as a reproducible platform-specific characteristic that warrants further investigation.

C-arm linacs and CyberKnife systems showed similar variations in passing rates. Because only two Radixact machines were included, no firm conclusions can be drawn for that subgroup. To contextualize these findings, we compared them with the only national stereotactic audit available to date [25]. The UK audit reported point-dose deviations at the PTV center ranging from – 1.3% to +3.9% for C-arm linacs and from +1.4% to +4.0% for CyberKnife systems. In our study, the corresponding ranges were – 1.0% to + 1.2% for CyberKnife systems and 4.1% to 4.3% for C-arm linacs, with mean deviations closer to zero (approximately – 0.2% for linacs and +0.5% for CyberKnife systems). These findings suggest slightly reduced variability and improved agreement across institutions in Switzerland, although differences in methodology, sample size, and the inclusion of repeated measurements in our intercomparison should be considered when interpreting these comparisons.

Gamma passing rates for both normalization methods and criteria were substantially higher than those reported by Dimitriadis et al., despite a similar inclusion of high- and low-dose regions. Ion chamber and film measurements showed excellent agreement, with <1% variation for 27 devices and within 2% for the remaining three, all within the combined uncertainty of the two detectors (1.35–1.8%), as derived from the study-specific uncertainty analysis detailed in Supplementary Table S2 and Table S3.

Although the PinPoint 3D detector is not ideal for very small fields due to volume-averaging and density effects [27], [29]., using a 1.5 cm diameter target and applying correction factors for a 1.5 × 1.5 cm^2^ field avoided significant systematic under-response in this study. These correction factors are planar, however, and suitable 3D corrections for dynamic techniques such as volumetric modulated arc therapy remain unknown. Air-to-water stopping-power ratios are approximately unity for composite and static fields. To our knowledge, only one study has evaluated ionization-chamber response under non-equilibrium conditions in modulated fields, suggesting that air-filled chambers may require similar small-field correction factors in composite and static fields [30]. Finally, differences in prescription conventions, particularly the inhomogeneous prescriptions used in CyberKnife, may contribute to inter-institutional variability despite chamber placement at the PTV center. A separate manuscript has addressed a detailed comparative analysis of planning and prescription practices across centers [21].

In this study, film dosimetry was limited to a single plane due to time constraints. The Prime phantom cannot irradiate orthogonal films simultaneously, which would require multiple sequential setups. Future intercomparisons may benefit from 3D dosimetry solutions or head phantoms that allow film inserts in two perpendicular planes.

A dose threshold of 2 Gy per fraction (10 Gy total) was applied to define the gamma analysis region, focusing on clinically relevant medium- to high-dose areas. Although no strict cutoff for clinical irrelevance exists, doses below 10 Gy are unlikely to produce meaningful toxicity in critical brain structures. The American Association of Physicists in Medicine (AAPM) HyTEC consensus recommends keeping normal brain V_24Gy_ < 20 cm^3^ to limit radionecrosis risk to <10%, with similar caution for V_20 Gy_ in hypofractionated regimens [31]. Organ-specific constraints for the brainstem and optic apparatus also lie in the low-20 Gy range (Task Group 101). Thus, the 10 Gy threshold remains well below established toxicity limits and supports meaningful evaluation of dosimetric accuracy in regions of stereotactic importance. Details of the film-specific methodological rationale are provided in Supplementary Material A2.

Another limitation of this study was the small number of Radixact and CyberKnife participants, which reduced the robustness of the statistical analysis. Addressing this limitation is inherently difficult in national-level intercomparisons, particularly for advanced stereotactic techniques. This study also focused exclusively on the dosimetric component of intracranial radiosurgery.

A comprehensive end-to-end intercomparison would require inclusion of magnetic resonance (MR) image quality and CT/MR fusion accuracy in future evaluations, as these components are recognized sources of geometric and dosimetric uncertainty in radiotherapy workflows [32], [33].

Despite the inherent complexity of stereotactic radiotherapy, most participating centers demonstrated clinically acceptable agreement within established tolerances, with only a small number of outliers. A few deviations in ion chamber or film measurements led several centers to request follow-up assessments. For the three centers whose results improved in the second measurement despite reporting no changes to their machines, we confirmed that these improvements did not stem from variability in our own measurement or analysis, as ion chamber and film dosimetry were consistent across both sessions. We therefore reported both initial and repeated results transparently, acknowledging that the cause of the improvement remains unclear. Although a third independent measurement might have provided further clarification, conducting full end-to-end dosimetric tests across multiple institutions requires substantial logistical, clinical, and financial resources and was not feasible within this intercomparison. Nevertheless, situations where results improved without any modification in delivery raise an important clinical concern: how can one be certain which dose a patient would actually receive? This highlights that while a single intercomparison provides valuable benchmarking, it may not fully capture day-to-day variability in stereotactic dose delivery. By transparently including both initial and repeated results, this study offers a realistic view of variability across institutions and reinforces the role of national intercomparisons in continuous clinical refinement and quality assurance.

This nationwide intercomparison, covering 90% of radiotherapy centers in Switzerland, provided a robust platform for evaluating the accuracy of stereotactic dose delivery and calculation under real-world clinical conditions. Similar large-scale national end-to-end audits have demonstrated the value of benchmarking in identifying inter-institution variability and supporting consistency across radiotherapy centers [34]. Beyond benchmarking, it fostered self-assessment and constructive comparison across diverse technologies and workflows. As stereotactic techniques evolve, such collaborative initiatives remain essential for maintaining high standards and strengthening national consistency in radiotherapy practice. Switzerland’s heterogeneous landscape—including C-arm linacs, CyberKnife systems, and Radixact systems with varied planning strategies—highlights the value of establishing a national baseline of dosimetric performance. While international audits demonstrate feasibility and rigor, this country-specific intercomparison offers directly actionable data for national QA initiatives, accreditation, and policy discussions. The novelty and impact of this work lie not only in the measurement methodology but also in establishing the first Swiss benchmark for stereotactic radiotherapy, ensuring comparability and safety across institutions and forming the foundation for future periodic audits.

## CRediT authorship contribution statement

**Sara Abdollahi:** Writing – review & editing, Writing – original draft, Visualization, Validation, Methodology, Investigation, Formal analysis, Data curation. **Rachid Boucenna:** Writing – review & editing, Investigation, Data curation. **Cécile Chatelain:** Writing – review & editing, Investigation, Data curation. **Nathan Corradini:** Writing – review & editing, Investigation, Data curation. **Marie Fargier-Voiron:** Writing – review & editing, Investigation, Data curation. **Vincent Fave:** Writing – review & editing, Investigation, Data curation. **Juan Garcia:** Writing – review & editing, Investigation, Data curation. **Sarah Ghandour:** Writing – review & editing, Investigation, Data curation. **Matthias Guckenberger:** Writing – review & editing, Validation. **Martin Härtig:** Writing – review & editing, Investigation, Data curation. **Tanja Hertel:** Writing – review & editing, Investigation, Data curation. **Maud Jaccard:** Writing – review & editing, Investigation, Data curation. **David Jeller:** Writing – review & editing, Investigation, Data curation. **Stephan Klöck:** Writing – review & editing, Investigation, Data curation. **Krayenbühl Jérôme:** Writing – review & editing, Validation, Supervision, Methodology, Formal analysis, Conceptualization. **Natacha Ruiz López:** Writing – review & editing, Investigation, Data curation. **Peter Pemler:** Writing – review & editing, Investigation, Data curation. **Harald Petermann:** Writing – review & editing, Investigation, Data curation. **Olivier Pisaturo:** Writing – review & editing, Investigation, Data curation. **Francesco Pupillo:** Writing – review & editing, Investigation, Data curation. **Daniel Schmidhalter:** Writing – review & editing, Investigation, Data curation. **Christian Tata:** Writing – review & editing, Investigation, Data curation. **Sheeba Thengumpallil:** Writing – review & editing, Investigation, Data curation. **Sergejs Unterkirhers:** Writing – review & editing, Investigation, Data curation. **Veronique Vallet:** Writing – review & editing, Investigation, Data curation. **Patrick Weber:** Writing – review & editing, Investigation, Data curation. **Nicolaus Andratschke:** Writing – review & editing, Validation, Supervision, Funding acquisition. **Stephanie Tanadini-Lang:** Writing – review & editing, Validation, Supervision, Funding acquisition, Formal analysis.

## Declaration of competing interest

The authors declare the following financial interests/personal relationships which may be considered as potential competing interests: The University Hospital Zurich holds research and teaching agreements with Varian/Siemens Healthineers, which may be considered a potential conflict of interest. However, these agreements had no influence on the design, execution, or interpretation of this study.
