# Supplementary material for: Dosimetric intercomparison for stereotactic radiotherapy of multiple brain metastases
Source: Phys Imaging Radiat Oncol. 2026 Mar 28;38:100956. doi: 10.1016/j.phro.2026.100956 (PMC13085090; doi:10.1016/j.phro.2026.100956)
Supplement: Supplementary Data 1 [file mmc1.pdf]

Supplementary Materials

A. Dosimetric Measurement Procedures

A.1. Ion chamber measurements

For ion chamber measurements, the phantom in each institution was set up on the treatment couch by aligning the crosshairs on the top and side of the phantom with the patient setup laser system. The position was then fine-tuned using the available image guidance localization technique at each department. The correct positioning of the ionization chamber's sensitive volume was verified in C-arm linacs and Radixact systems using registration to bony anatomy; however, such verification was not feasible for CyberKnife machines, which rely on planar imaging. Point-dose measurements were performed with a PTW PinPoint 3D ion chamber and PTW UNIDOS webline electrometer. Chamber readings were corrected for temperature, pressure, and beam quality at each department.

The TRS-483 output correction factors ($k_{Q_{clin},fQ_{msr}}^{f_{clin},f_{msr}}$) for a 1.5 × 1.5 cm² field with the PinPoint 3D ion chamber (1.013 for C-arm Linacs and Radixact, 1.011 for CyberKnife) were applied to all measurements [1]. We note that only one published study [2] has proposed estimating plan-specific equivalent field sizes for modulated deliveries by analysing individual control points or MU-weighted apertures. While such methods can be informative, they are highly plan-dependent and are not included in current small-field dosimetry guidance, including IAEA TRS-483. Because centre-specific dynamic corrections would reduce robustness and comparability in a national intercomparison, we applied the TRS-483 correction factor corresponding to the nominal PTV diameter (1.5 cm), ensuring a consistent and reproducible methodology across all institutions.

The measured dose was then compared with the calculated mean dose in the chamber's sensitive volume for each institution. The combined standard uncertainty (k = 1) associated with PinPoint 3D dosimetry was estimated at 1.35%, with contributing elements listed in Table S.1 of the Supplementary Materials. Although scintillator detectors such as microDiamond provide near-unity correction factors in very small fields, they are more sensitive to density perturbation effects in the radial irradiation geometry used for plan verification, leading to a slight over-response. In contrast, the PinPoint 3D ion chamber exhibits a small under-response in this geometry but with lower directional dependence and greater robustness, making it more suitable for multi-institutional audits. By defining the chamber target with a 1.5 cm diameter, the under-response of the PinPoint 3D due to volume averaging was minimized, ensuring reliable measurements within the uncertainty of the study.

In addition to detector selection, the reliability of our reference dosimetry was independently assured. The host center regularly participates in external dosimetry verification programs, including the IROC Houston (MD Anderson) optically stimulated luminescence dosimeter (OSLD) intercomparison and the Swiss Society for Radiation Oncology Medical Physics (SSRMP) national thermoluminescent dosimeter TLD audit, with results consistently within accepted tolerance limits. Furthermore, the PinPoint 3D ion chamber used in this study was cross-calibrated against our departmental reference chamber, which in turn is calibrated at the Swiss Federal Institute of Metrology (METAS), ensuring traceability to national standards.

A.2. Film measurements

In ensuring accurate film dosimetry with EBT3 films, the measurement were performed for single fraction of 6 Gy. PTV1 (1.5 cm) was measured using both the ionization chamber (IC) and film, while PTV2 (1 cm) was measured using film only. PTV1 and PTV2 were located in the same coronal plane, approximately 7 cm apart, which allowed us to place the film in that plane to simultaneously evaluate both targets. PTV3 (0.5 cm), which was positioned 4 cm superior to PTV1 in the axial direction, was not included in the dose measurements. This target was included to represent a more complex, non-coplanar lesion geometry, which is commonly encountered in stereotactic radiotherapy of multiple brain metastases. Although we did not perform direct dose measurements for PTV3, its inclusion allowed us to evaluate the treatment planning system's capability to simultaneously plan and optimize dose distributions for multiple targets with varying spatial relationships. This reflects realistic clinical scenarios and contributes to the overall assessment of plan quality and consistency across institutions.
Phantom localization was performed using each department's routine stereotactic workflow: kilovoltage cone beam CT (kV CBCT) on C-arm linacs, kVCT or megavoltage CT (MVCT) on Radixact systems, and stereoscopic kV imaging on CyberKnife. The setup imaging dose from kV CBCT and MVCT was not corrected for, as its contribution is negligible relative to the 6 Gy treatment dose delivered in this study.
All measurements were conducted by the same individual (the USZ representative) in collaboration with the medical physicists at each host institution. This ensured consistent measurements throughout the study. The film response was calibrated at USZ with eight EBT3 films over the range of 0–10 Gy using a 6 MV linear accelerator, with a traceable calibration to the Federal Institute of Metrology (METAS). Gafchromic EBT3 film was selected for this study because of its high spatial resolution, near water-equivalence, and minimal dependence on energy and dose rate. These characteristics make it particularly well suited for stereotactic applications, where accurate dose verification in regions with steep dose gradients is essential. The films were irradiated in a 10 cm × 10 cm field size using 6 MV beam in 90 cm Source to skin distance (SSD) and 10 cm depth in RW3 slab phantom. An Epson Expression 12000XL flat-bed scanner (Seiko Epson Corp, Nagano, Japan) was used to scan the calibration and plan quality assurance films in red–green–blue format [3, 4] with 150 dots per inch using transmission mode with no corrections. Films were placed in the center of the scanner bed to have better scanner response uniformity. A clear glass plate was placed over the films to reduce the dose uncertainty due to the Callier effect [5, 6]. The average pixel value in a 1.5 × 1.5 cm region at the center of the beam axis was used to calculate the film calibration curve using FilmQAPro® software. Film calibration was performed using the rational quadratic calibration model implemented in FilmQAPro software. The model follows $X(D) = \frac{P_2\, D}{D+E}$, where D is absorbed dose and the parameters P_2 and E are obtained from the calibration measurements. This form accurately models the nonlinear response of Gafchromic EBT3 films over the 0–10 Gy range, particularly addressing the flattening behavior at higher doses. The fitting process aimed to keep the root-mean-square residual error below 1.3%, ensuring high calibration accuracy without overfitting. Only a limited number of fitting parameters were used, and the model complexity is minimal and physically justified by the known characteristics of radiochromic film response. Furthermore, during the film analysis, no auto-alignment, best-fit, or shifting algorithms were applied during the gamma analysis. The agreement between the calculated and measured film dose distributions was based on the initial mechanical alignment of the film using the central mark of the phantom. The planned isocenter was registered with the film central mark in the FilmQAPro® software, and all analyses were performed without

any post-processing adjustment. Dose maps were generated from the images by applying the red-green-blue triple-channel dosimetry algorithm in the software [4]. The triple-channel dosimetry algorithm was employed to generate accurate dose maps by combining information from the red, green, and blue channels, thereby correcting for film non-uniformities, scanner response variations, and lateral artifacts during calibration and dose mapping. However, for gamma analysis, only the red channel was used, as it provides the highest sensitivity and lowest noise for the dose range used in this study, which is standard practice in film dosimetry. This approach allows for more reliable and reproducible gamma index evaluation while still benefiting from the enhanced calibration and correction provided by the triple-channel processing.
In addition, no explicit normalization was applied to the film dose distributions. Instead, we decided not to correct for potential variations in linac output or film response during scanning. These sources of variability were accounted for in the overall uncertainty budget. This approach allowed us to preserve the integrity of the raw film measurements while transparently acknowledging and incorporating the possible deviations in dose delivery and film readout into the analysis. Moreover, applying normalization could have artificially reduced apparent discrepancies, especially in small target dosimetry. By analysing the films in absolute terms, we preserved sensitivity to both systematic and random deviations across centers. The agreement between the planned dose and the measured dose was assessed and gamma passing rates were calculated using the red color channel and various criteria including global and local assessments, employing gamma criteria of 5%/1 mm and 3%/1 mm.
Gamma passing rate alone lacks clinical specificity, as the percentage of points meeting gamma criteria is highly sensitive to analysis parameters such as the region of interest or dose threshold. Previous studies have highlighted the limitations of gamma analysis for dose distribution comparisons [7, 8].
Nevertheless, gamma analysis remains one of the most widely used methods in clinical practice. By appropriately defining the region of interest and dose cutoff to include both high and low dose areas, this analysis successfully evaluated the dose delivery performance of all SRT platforms included in the study. Given these limitations, we complemented the gamma analysis with independent point-dose verification. Film dosimetry itself provided insight into both positional accuracy and dose agreement through direct comparison of measured and planned profiles. Ion chamber measurements were therefore included not as the sole orthogonal metric, but as a quantitative double-check to confirm and strengthen the findings from the film analysis. Together, these approaches allowed us to distinguish whether high gamma pass rates reflected true dose calculation accuracy or were primarily driven by mechanical and positional precision.

In this study, a cutoff threshold of 2 Gy was applied for local gamma analysis to exclude low-dose regions with high film uncertainty. While task group-218 recommends a 10% threshold for global gamma analysis, no explicit guidance is provided for local gamma. A recent systematic review by Malatesta et al. [9] highlighted the variability of patient-specific quality assurance (PSQA) methodologies in stereotactic body radiotherapy (SBRT), including differences in gamma criteria and cutoff definitions, and emphasized the need for transparent reporting. In line with these recommendations, we report our use of a 2 Gy local cutoff—above the reliable film response range of 1–1.5 Gy and corresponding to ~23–29% of the maximum dose in our plans—together with the 10% global cutoff, ensuring clarity, reproducibility, and a focus on clinically relevant dose regions. The broader clinical rationale for this cutoff is discussed in the main text (Discussion).

Table S.1. Summary of treatment devices, techniques and treatment plans of different institutions. (VAR=Varian, ELK=Elekta, ACC=Accuray, CK=Cyberknife, RAD= Radixact, FFF= flattening filter free beam,

FL= flat beam, Ac= Acuros, CCC= Collapse Cone Convolution, MC= Monte Carlo, RT= Ray Tracing, FCBB= Fluence-Convolution Broad-Beam)

**Device No.**	**Treatment Device**	**MLC leaf width**	**Energy**	**Technique**	**Coplanar/ Non-cop.**	**TPS**	**Dose Calc. Algorithm**	**Treat. Time***
1	ACC Radixact	6.25 mm	6FFF	2 Iso.	Coplanar	Ray Station	CCC	4.9min
2	VAR True Beam	5 mm	6FFF	One Iso. 3Arcs	Non-cop. 2couch kicks	Eclipse	Ac	2.5min
3	VAR True Beam	5 mm	6FFF	One Iso. 3Arcs	Non-cop. 2couch kicks	Eclipse	Ac	2.5min
4	VARClinac-iX	5 mm	6FFF	3 Iso. 3Arcs.	Coplanar	Eclipse	Ac	3min
5	VAR True Beam	5 mm	6FFF	2 Iso. 8Arcs	Non-cop.	Eclipse	Ac	6min
6	VAR True Beam STx	2.5 mm	10FFF	One Iso. 8Arcs	Non-cop. 4couch kicks	Eclipse	Ac	5.3min
7	VAR True Beam	5 mm	6FFF	2 Iso. 4Arcs	Non-cop.	Eclipse	Ac	2min
8	ACC CyberKnife	Cone (7.5,10,12.5,15)	6FFF	One Plan, 72nodes, 142beams	Non-cop.	Precision	RT	42min
9	VAR True Beam	5 mm	6FFF	2 Iso. 10Arcs.	Non-cop.	Eclipse	Ac	6.6min
10	VAR Edge	2.5 mm	6FFF	2 Iso. 6Arcs.	Non-cop. 4couch kicks	Eclipse	Ac	5.3min
11	VAR Edge	2.5 mm	6FFF	2 Iso. 6Arcs.	Non-cop. 4couch kicks	Eclipse	Ac	5.3min
12	VAR True Beam	5 mm	6FFF	2 Iso. 11Arcs.	Non-cop. 11couch kicks	Eclipse	Ac	6.1min
13	VARClinac-iX	2.5 mm	6FFF	2 Iso. 9Arcs.	Non-cop. 4couch kicks	Ray Station	CCC	9min
14	ELK Versa HD	5 mm	6FFF	One Iso. 8Arcs	Coplanar	Ray Station	MC	9.3min
15	VAR True Beam	5 mm	6FFF	3 Iso. 15Arcs.	Non-cop. 5couch kicks	Eclipse	Ac	12min
16	ACC CyberKnife S7	Iris (7.5,10,12.5,15)	6FFF	One Plan, 117nodes, 184beams	Non-cop.	Precision	RT	41min
17	ACC CyberKnife M6	Cone (7.5,12.5)	6FFF	One Plan, 108nodes, 206beams	Non-cop.	Precision	RT	56min
18	VAR True Beam	5 mm	6FFF	3 Iso. 13Arcs.	Non-cop.	Eclipse	Ac	13min
19	VAR True Beam	5 mm	6FFF	2 Iso. 4Arcs.	Coplanar	Eclipse	Ac	4min
20	ELK Synergy	5 mm	6FFF	3 Iso. 3Arcs.	Coplanar	Pinnacle	CCC	3min
21	VAR True Beam	5 mm	6FFF	2 Iso. 6Arcs	Non-cop. 2couch kicks	Eclipse	Ac	6min
22	VAR True Beam	5 mm	6FFF	One Iso. 4Arcs	Non-cop.	Eclipse	Ac	2.5min
23	ACC CyberKnife S7	Iris (10,12.5)	6FFF	One Plan, 97nodes, 153beams	Non-cop.	Ray Station	MC	39min
24	VAR Edge	2.5 mm	6FFF	One Iso. 3Arcs	Non-cop. 2couch kicks	Eclipse	Ac	2.5min
25	ACC CyberKnife M6	Iris (10,12.5,15)	6FFF	One Plan, 79nodes, 149beams	Non-cop.	Ray Station	MC	25min
26	ELK Synergy	5 mm	6WFF	3 Iso. 11Arcs.	Non-cop. 6couch kicks	Monaco	MC	12min
27	VAR Edge	2.5 mm	6FFF	2 Iso. 10Arcs	Non-cop.	Eclipse	Ac	8.3min
28	VAR Edge	2.5 mm	10FFF	One Iso. 4Arcs	Non-cop.	Eclipse	Ac	2min
29	VAR True Beam	5 mm	6FFF	One Iso. 4Arcs	Non-cop.	Eclipse	Ac	2.5min
30	ACC Radixact	6.25 mm	6FFF	2 Iso.	Coplanar	Precision	CCCS/FCBB	9min

*The treatment times presented in the table were calculated based on beam-on time for C-arm linacs and Radixact systems. However, for CyberKnife machines, the reported times include both robot movement and imaging performed during treatment.

B. Measurement repetition

The repeated measurements involved two CyberKnife systems, two Radixact systems, one Elekta linac, and two Varian linacs. For one Varian linac the measurement was repeated because of a wrong delivery in the first measurement. The measurement in the second Varian linac was repeated as it showed a point dose deviation of 7.6% in chamber measurement. It was then realized that the discrepancy was due to considering a wrong dose for chamber sensitive volume in the TPS rather than inaccuracy in dose delivery.
For one CyberKnife system, the initial film dosimetry showed a significant underdosage in both targets, while the ion chamber measurement indicated less than 2% deviation between the planned and delivered dose. This discrepancy likely stemmed from an issue intrinsic to the film dosimetry process or film response variability.
Regarding the local accuracy, film measurement showed 0.6 mm positional shift in both longitudinal and lateral directions for both plans separately design for superior and inferior targets. The second measurement was asked from the hospital to investigate any measurement error leading to a positional shift and the dose deviation. In the second measurement, they changed their planning method from three plans for three targets to one plan for all targets. The second measurement reproduced the same minor positional shifts but demonstrated dose agreement within expected tolerances, effectively ruling out delivery errors and confirming that the initial dose deviation was attributable to a film-related issue.
The film measurement results for the second CyberKnife system showed positional inaccuracies in some specific directions of dose delivery, and it completely resolved in the second measurement. It was stated by the hospital that no changes or maintenance has been performed between the first and the second measurement.
For one Radixact system, calculated dose was 6.5% lower than the measured dose in the first measurement and the EBT3 film measurement showed 5.5% dose difference in the position of chamber sensitive volume. The ion chamber and film dose measurement results were improved to 4.3% and 3% dose discrepancies in the consecutive measurement. This is while hospital reported dose difference of 1.6% with their PinPoint 3D chamber. It was stated by the hospital that no changes or maintenance has been performed between the first and the second measurement. For the other Radixact machine, calculated dose was 7.7 % lower than the measured dose in the first measurement and the EBT3 film measurement showed 7.2% dose difference in the position of chamber sensitive volume. The ion chamber and film dose measurement results were improved to 4.3% and 3.5% dose discrepancies in the consecutive measurement. The phantom localization was performed with MVCT instead of kVCT in the second measurement.
Regarding the Elekta linac, the hospital asked for the second measurement as the first results showed 6.3% dose deviation in point dose measurement when the EBT3 film measurement also showed 5.8% dose difference in the position of chamber sensitive volume. The main deviation was probably due to the positional shifts of about 0.6 mm in both lateral and longitudinal directions. The center opted to modify their plan by substituting couch rotation with no rotation during the second measurement. This adjustment was aimed at optimizing the alignment between the delivered and the planned dose. In the second measurement the results were improved with 2.6% dose deviation in point dose measurement and lateral misalignment was solved while longitudinal was observed again.

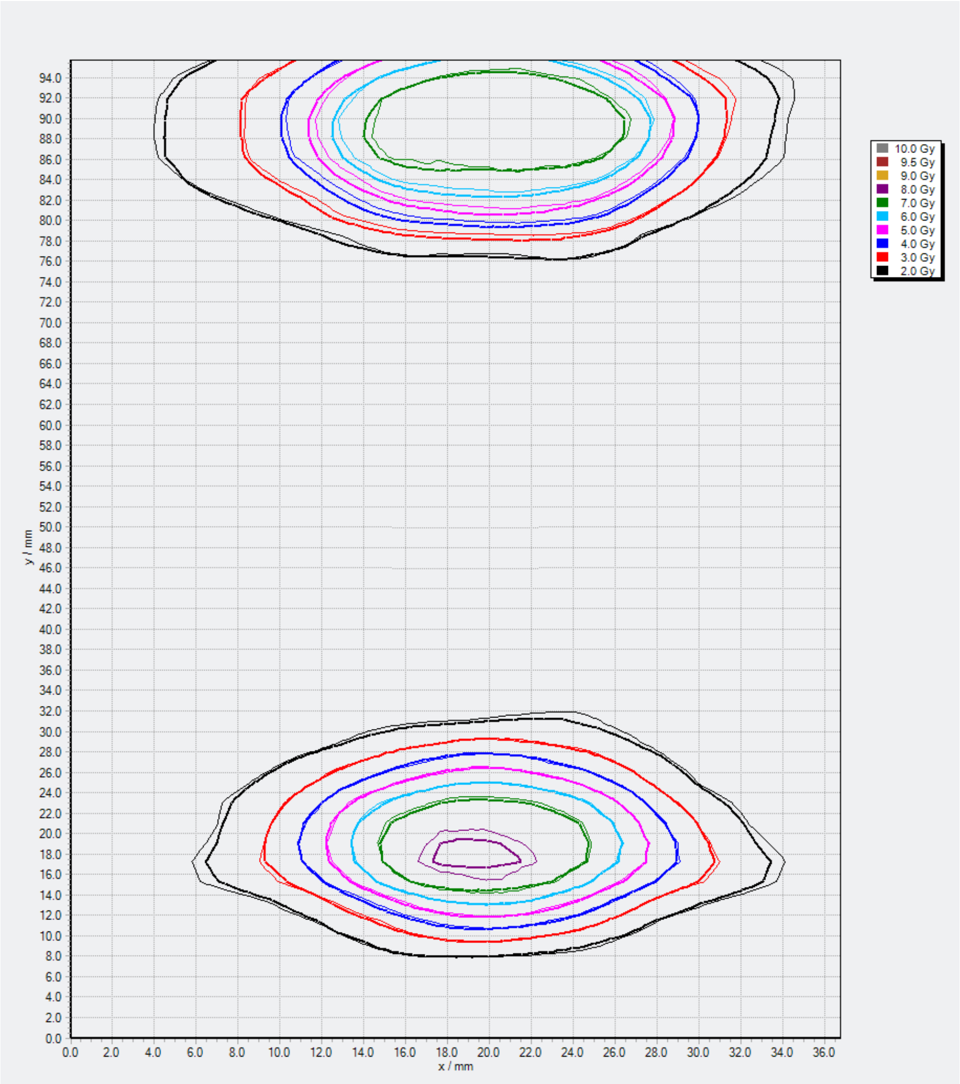

Figure S.1. A sample for the dose distribution comparisons between the film-measured doses (thin lines) and the treatment planning system calculated doses (thick lines) for superior and inferior targets.

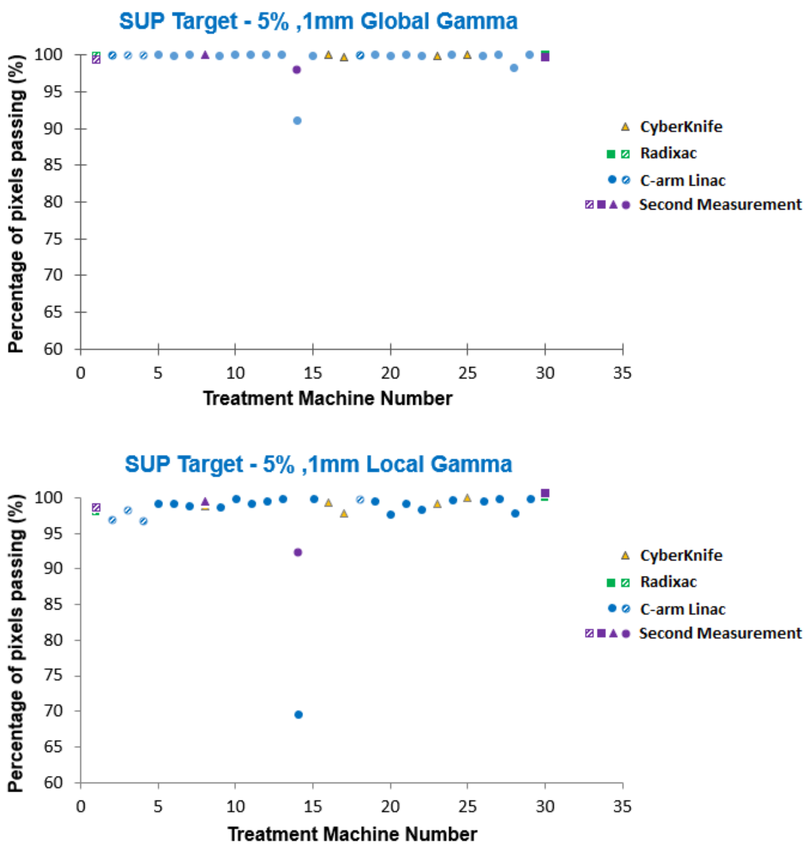

Figure S.2. Gamma passing rates for superior target (5%, 1 mm global and local criteria) across treatment platforms. Purple markers denote second measurements, while pattern-filled markers indicate systems not commissioned for SRS/SRT.

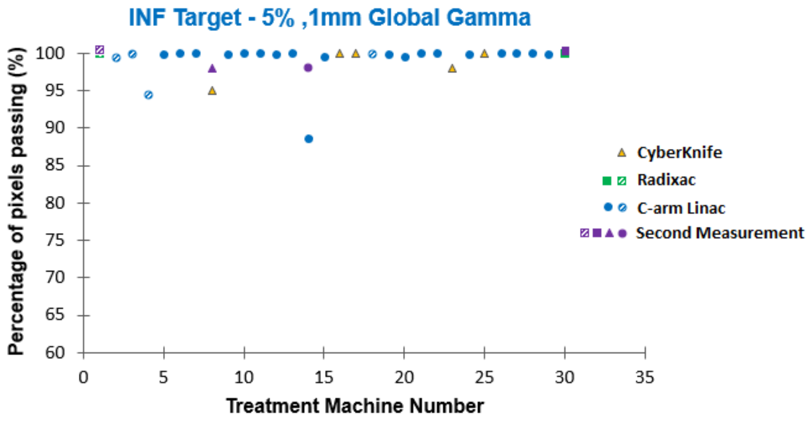

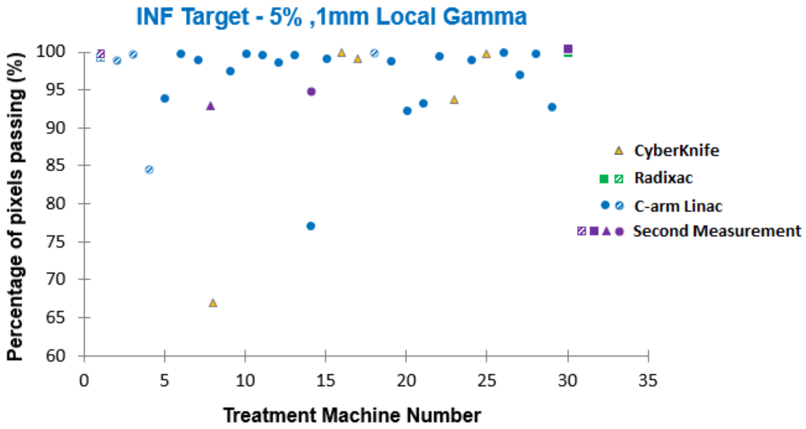

Figure S.3. Gamma passing rates for inferior target (5%, 1 mm global and local criteria) across treatment platforms. Purple markers denote second measurements, while pattern-filled markers indicate systems not commissioned for SRS/SRT.

C. Statistical Analysis Methods and Results

The Kruskal-Wallis test was performed to compare Radixact, C-Arm Linacs, and CyberKnife, using gamma pass rates with various normalizations (global and local) and different gamma criteria (5%, 1mm and 3%, 1mm) for both superior and inferior targets. Additionally, this test was applied to compare the gamma pass rate for film analysis across four different dose calculation algorithms (Monte Carlo, Acuros XB, Collapse Cone Convolution, and Ray Tracing), coplanar and non-coplanar plans, and plans combining all targets in a single plan versus separate plans for each target.

C.1. Ion Chamber Measurements

The Kruskal-Wallis test revealed a significant difference among the three groups ($p = 0.00008$). Pairwise Mann-Whitney U tests showed significant differences between Radixact and Linacs ($p = 0.0062$), and between Radixact and CK ($p = 0.0286$), but not between Linacs and CK ($p = 0.486$). A Bonferroni correction was applied to adjust the significance level for multiple pairwise comparisons, ensuring control of the family-wise error rate.

C.2. Film Measurements

The Kruskal-Wallis test among the three treatment platforms revealed a statistically significant difference only in the local gamma pass rate using the 3%, 1 mm criterion ($p = 0.0073$). Subsequent pairwise comparisons with the Mann-Whitney U test showed that the Radixact group outperformed the C-Arm Linacs group ($p = 0.0047$), while no significant differences were found between Radixact and CyberKnife ($p = 0.146$) or between C-Arm Linacs and CyberKnife ($p = 0.112$).

No statistically significant differences in gamma pass rates were observed among the dose calculation algorithms. For the superior target, the Kruskal-Wallis test returned $p = 0.0721$ (5%, 1 mm) and $p = 0.7848$ (3%, 1 mm). For the inferior target, the results were similarly non-significant: $p = 0.7424$ (5%, 1 mm) and $p = 0.5097$ (3%, 1 mm), indicating that the algorithm choice did not significantly affect gamma pass rates.

Comparisons between single- and multiple-isocenter plans also showed no significant differences across all evaluated criteria. For the inferior target, the p-values were 0.877 (local gamma, 3%, 1 mm), 0.768 (local gamma, 5%, 1 mm), 0.340 (global gamma, 3%, 1 mm), and 0.225 (global gamma, 5%, 1 mm). For the superior target, the results were $p = 0.122$ (local gamma, 3%, 1 mm), $p = 0.210$ (local gamma, 5%, 1 mm), $p = 0.054$ (global gamma, 3%, 1 mm), and $p = 0.228$ (global gamma, 5%, 1 mm).

Finally, gamma pass rate comparisons between coplanar and non-coplanar plans revealed no statistically significant differences. For the inferior target, the p-values were 0.481 and 0.293 (local gamma, 3% and 5%, 1 mm), and 0.370 and 0.256 (global gamma, 3% and 5%, 1 mm). For the superior target, local gamma comparisons yielded $p = 0.179$ (3%, 1 mm) and $p = 0.174$ (5%, 1 mm), while global gamma results were $p = 0.495$ (3%, 1 mm) and $p = 0.514$ (5%, 1 mm). These findings indicate that beam geometry—coplanar versus non-coplanar—did not have a statistically significant effect on gamma pass rates under either local or global criteria.

Table S.2. Uncertainty budget for PinPoint3D dosimetry in 6 MV FFF beam (%) (k=1)

Step 1: Calibration of the reference dosimeter at the PSDL (METAS)	1.05
$N_{D,W}$ calibration of the reference dosimeter (PTW 30013 Farmer) in FFF beam [1,10]	1.05
Step 2: Cross-calibration of the field dosimeter (PTW 30016 PinPoint3D)	0.55
Electrometer Reading [10]	0.29
Reproducibility (A)	0.03
Resolution (B)	0.01
Linearity (B)	0.03
Zero (B)	0.01
Long-term stability of the reference dosimeter (Farmer PTW 30013) (B)	0.29
Leakage (B)	0.01
Establishment of the reference conditions [11]	0.47
Positioning of the chamber at the reference depth (± 0.5 mm, 1 % / mm)	
Positioning of the chamber at SSD (1 mm)	
Step 3: Absorbed dose measurement in the user's beam	0.65
Corrected Reading [11]	0.45
Electrometer Reading:	0.29
Reproducibility (A)	0.03
Resolution (B)	0.01
Linearity (B)	0.03
Zero (B)	0.01
Long-term stability of the reference dosimeter (Farmer PTW 30013) (B)	0.29
Leakage (B)	0.01
Correction Factors:	0.34
Stability in dosimeter reading (relative to external monitor)	0.14
Temperature	0.18
Pressure	0.09
Humidity	0.1
Correction for recombination effect	0.16
Correction for polarity effect	0.14
Establishment of the reference conditions [11]	0.47
Positioning of the chamber at the reference depth (± 0.5 mm, 1 % / mm)	
Positioning of the chamber at SSD (1 mm)	
**Combined standard uncertainty (k=1)**	**1.35**

Table S.3. Uncertainty budget for Gafchromic EBT3 film dosimetry (%) (k=1)

Calibration of the linear accelerator output for reference film exposures	1.23
$N_{D,W}$ calibration of the reference dosimeter (PTW 30013 Farmer) (METAS)	0.9
Corrected Reading [11]	0.45
Electrometer Reading:	0.29
Reproducibility (A)	0.03
Resolution (B)	0.01
Linearity (B)	0.03
Zero (B)	0.01
Long-term stability of the reference dosimeter (Farmer PTW 30013) (B)	0.29
Leakage (B)	0.01
Correction Factors:	0.34
Stability in dosimeter reading (relative to external monitor)	0.14
Temperature	0.18
Pressure	0.09
Humidity	0.1
Correction for recombination effect	0.16
Correction for polarity effect	0.14
Establishment of the reference conditions [11]	0.47
Positioning of the chamber at the reference depth (± 0.5 mm, 1 % / mm)	
Positioning of the chamber at SSD (1 mm)	
Calibration curve fit of the film response function	1.3
Film non-uniformity [12]	0.2
Film scanning process [13], *	0.1
**Combined standard uncertainty (k=1)**	**1.8**

*Film orientation and film position with respect to the scanner were kept consistently.

Supplementary References

[1] IAEA. Dosimetry of small static fields used in external beam radiotherapy. Technical Reports Series No. 483. Vienna: International Atomic Energy Agency; 2017. Available from: https://www.iaea.org/publications/11075/dosimetry-of-small-static-fields-used-in-external-beam-radiotherapy

[2] Desai VK, Labby ZE, Hyun MA, DeWerd LA, Culberson WS. VMAT and IMRT plan-specific correction factors for linac-based ionization chamber dosimetry. Med Phys 2019;46:913-24. https://doi.org/10.1002/mp.13293

[3] Pérez Azorín JF, Ramos García LI, Martí-Climent JM. A method for multichannel dosimetry with EBT3 radiochromic films. Med Phys 2014; 41:062101. https://doi.org/10.1118/1.4871622

[4] Méndez I, Polšak A, Hudej R, Casar B. The Multigaussian method: a new approach to mitigating spatial heterogeneities with multichannel radiochromic film dosimetry. Phys Med Biol 2018; 63:175013. https://doi.org/10.1088/1361-6560/aad9c1

[5] Niroomand-Rad A, Chiu-Tsao ST, Grams MP, Lewis DF, Soares CG, van Battum LJ, et al. Report of AAPM Task Group 235 radiochromic film dosimetry: an update to TG-55. Med Phys 2020; 47:5986-6025. https://doi.org/10.1002/mp.14497

[6] Palmer AL, Bradley DA, Nisbet A. Evaluation and mitigation of potential errors in radiochromic film dosimetry due to film curvature at scanning. J Appl Clin Med Phys 2015; 16:425-31. https://doi.org/10.1120/jacmp.v16i2.5141

[7] Miften M, Olch A, Mihailidis D, Moran J, Pawlicki T, Molineu A, et al. Tolerance limits and methodologies for IMRT measurement-based verification QA: recommendations of AAPM Task Group 218. Med Phys 2018; 45:53-83. https://doi.org/10.1002/mp.12810

[8] Ezzell GA, Burmeister JW, Dogan N, LoSasso TJ, Mechalakos JG, Mihailidis D, et al. IMRT commissioning: multiple institution planning and dosimetry comparisons, a report from AAPM Task Group 119. Med Phys 2009; 36:5359-73. https://doi.org/10.1118/1.3238104

[9] Malatesta T, Scaggion A, Giglioli FR, Belmonte G, Casale M, Colleoni P, et al. Patient specific quality assurance in SBRT: a systematic review of measurement-based methods. Phys Med Biol 2023; 68:21TR01. https://doi.org/10.1088/1361-6560/acf43a

[10] Budgell G, Brown K, Cashmore J, Duane S, Frame J, Hardy M, et al. IPEM topical report 1: guidance on implementing flattening filter free (FFF) radiotherapy. Phys Med Biol 2016; 61:8360. https://doi.org/10.1088/0031-9155/61/23/8360

[11] Castro P, García-Vicente F, Mínguez C, Floriano A, Sevillano D, Pérez L, et al. Study of the uncertainty in the determination of the absorbed dose to water during external beam radiotherapy calibration. J Appl Clin Med Phys 2008; 9:70-86. https://doi.org/10.1120/jacmp.v9i1.2676

[12] Marroquin EY, Herrera Gonzalez JA, Camacho Lopez MA, Barajas JE, García-Garduño OA. Evaluation of the uncertainty in an EBT3 film dosimetry system utilizing net optical density. J Appl Clin Med Phys 2016; 17:466-81. https://doi.org/10.1120/jacmp.v17i5.6262

[13] Sorriaux J, Kacperek A, Rossomme S, Lee JA, Bertrand D, Vynckier S, et al. Evaluation of Gafchromic EBT3 films characteristics in therapy photon, electron and proton beams. Phys Med 2013; 29:599-606. https://doi.org/10.1016/j.ejmp.2012.10.001
